# An Unusual Complication Seen in a Six-Year-Old Girl Treated with Open Reduction and Pemberton Osteotomy for Neglected Developmental Dysplasia of the Hip: A Femoral Neck Fracture Sustained during Passive Motion under General Anesthesia

**DOI:** 10.1155/2014/804098

**Published:** 2014-05-26

**Authors:** Vedat Uruc, Samet Karabulut

**Affiliations:** ^1^Department of Orthopedics and Traumatology, Medicine Faculty of Mustafa Kemal University, Antakya, Hatay, Turkey; ^2^Department of Orthopedics and Traumatology, Ergani State Hospital, Diyarbakır, Turkey

## Abstract

Despite the screening programs for newborn children with hip ultrasonography, neglected developmental dysplasia of the hip (DDH) is still continuing to be a problem in the east and southeast parts of our country. The main complications are redislocation, avascular necrosis, and joint stiffness. We present an unusual complication, femoral neck fracture during passive motion under general anesthesia, of a six-year-old girl with neglected DDH treated by open reduction and Pemberton osteotomy without femoral shortening. The fracture was treated by open reduction and internal fixation combined with proximal femoral shortening. After 5 years the patient had excellent clinical results, no avascular necrosis was seen, and the radiologic appearance was type IA according to modified Severin classification. In conclusion older children with neglected DDH are more likely to have joint stiffness after open reduction. If there is even a little doubt about joint stiffness after open reduction, one should not refrain from femoral shortening. Also passive motion under general anesthesia should be applied very carefully with fluoroscopic control.

## 1. Introduction

Currently DDH has almost disappeared in the developed countries due to the hip ultrasounds screening programs in the newborn. But unfortunately, in our country, especially in eastern and southeastern regions, it is still possible to come across children with neglected DDH. There are various surgical procedures reported in the literature for the treatment of DDH [1–7]. The treatment differs with the severity of dislocation, age, and surgeon experience. Surgical procedures include open reduction, femoral osteotomies, pelvic osteotomies, or combined procedures [2–4]. Femoral head avascular necrosis, redislocation, joint stiffness, graft fracture, and graft or pin displacements are the main complications reported in the literature [8–14]. Less frequently pin migration, superficial and deep wound infections, heterotopic ossification, and rotational deformity are also reported [15–17]. In this paper we presented an unusual complication during the treatment of a six-year-old girl with neglected DDH. Open reduction and Pemberton osteotomy were initially performed. Two months after surgery we removed the spica cast under general anesthesia. Moderate joint stiffness was seen. Passive motion was applied to improve the hip range of motion. Postoperative roentgenograms revealed femoral neck fracture. In our knowledge this complication has not been reported previously.

## 2. Case Report

**Figure 1:**
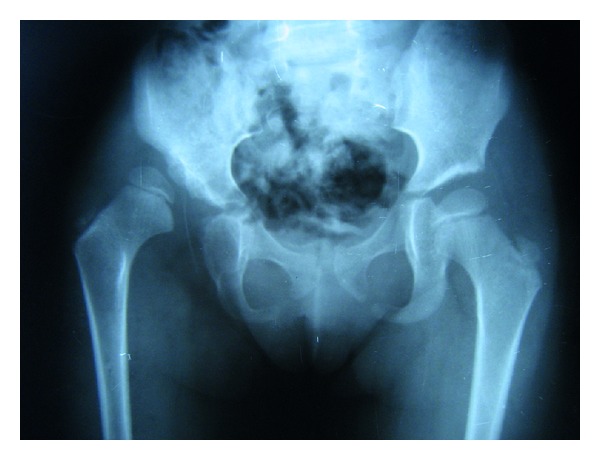
A six-year-old girl with right side neglected modified Severin classification type IV developmental dysplasia of hip.

**Figure 2:**
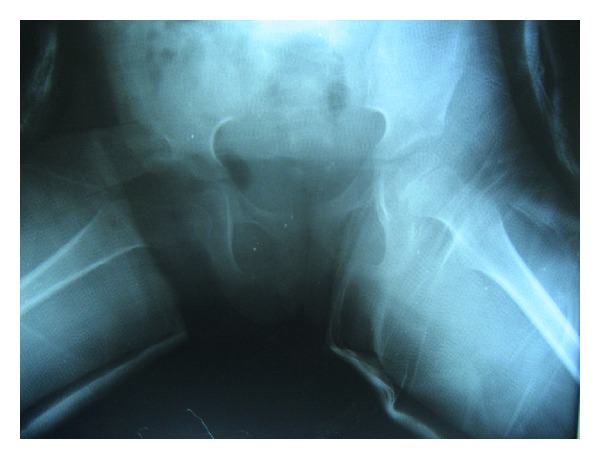
Plain radiograph after open reduction and Pemberton osteotomy.

**Figure 3:**
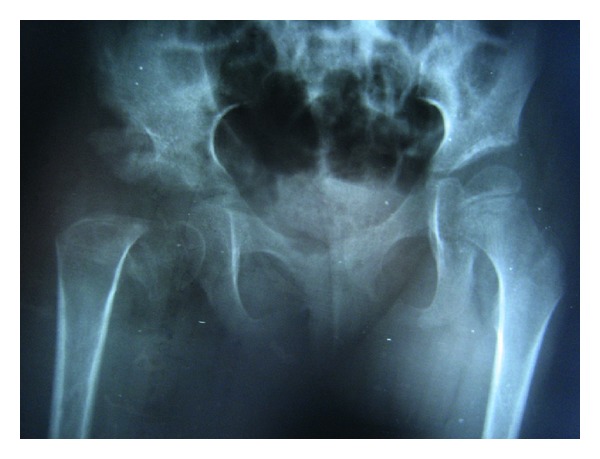
Right femoral neck fracture after passive motion under general anesthesia.

**Figure 4:**
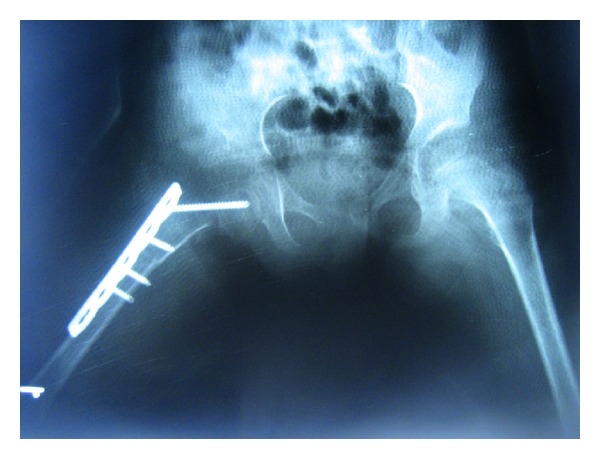
Revision surgery with open reduction and internal fixation of the femoral neck fracture combined with femoral shortening and derotation osteotomies.

**Figure 5:**
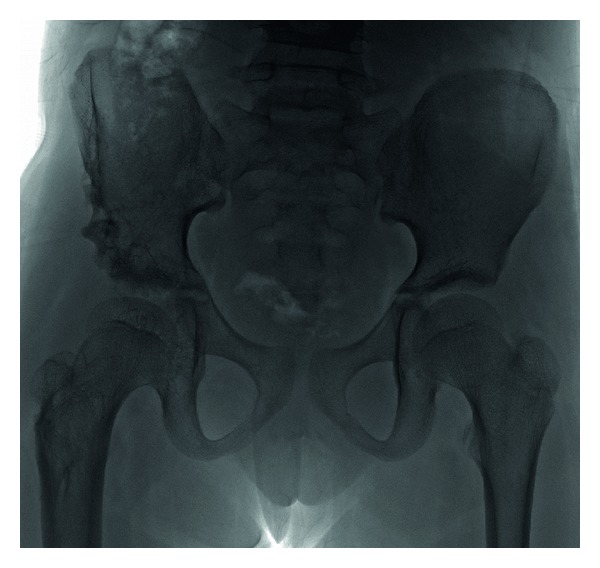
Control plain radiograph, five years after surgery.

A 6-year-old girl was admitted to our outpatient clinic with complaints of limping in June 2009. Clinical evaluation was fair according to modified McKay classification [8, 18]. She had a stable, painless hip, positive Trendelenburg sign, two cm shortening of right lower extremity, and limitation of abduction (30 degree). The roentgenograms revealed subluxation of the right hip (modified Severin type IV [19]) (Figure 1). The acetabular index measured 40 degrees. She was treated surgically; percutaneous adductor tenotomy, open reduction, and Pemberton osteotomy were done. Finally pelvipedal cast was applied. Postoperative roentgens were satisfactory (Figure 2). Two months after surgery the spica cast was removed under general anesthesia. Mild joint stiffness was present and passive motion was applied under general anesthesia to improve the joint motion. A femoral neck fracture was seen in the postoperative roentgenograms (Figure 3). It was stabilized by open reduction and internal fixation with a 4.5 mm narrow LC-DCP plate. Two centimeters femoral shortening and 30 degree derotation were also added to prevent the possible postoperative joint stiffness (Figure 4). Hip spica cast was applied for two months. We removed the spica cast at the end of two months. The fracture healing and hip reduction were satisfactory, but mild to moderate joint stiffness was present. She took physical therapy for one month. After six months, the implants were removed. The last control was done five years after surgery. The clinical evaluation was excellent according to the modified McKay classification [8, 18]: stable painless hip, no limping, no Trendelenburg sign, mild joint stiffness (flexion 120 degree, internal rotation 30 degree, external rotation 40 degree, and abduction 25 degree), and no leg length discrepancy. The control roentgenograms were excellent (type IA) according to modified Severin classification system [19], and CE angle measured 28 degrees (Figure 5). No avascular necrosis was seen, according to Bucholz and Ogden classification system [20]. The reduction was concentric (Figure 5).

## 3. Discussion

There is not a standard surgical procedure in the treatment of neglected DDH in older children. Treatment of neglected DDH is difficult due to adaptive shortening of the surrounding soft tissues, acetabular dysplasia, angulation, and rotation of the proximal femur [11]. The main purpose is to obtain a concentric reduced and stable hip. Proximal femoral varization-derotation osteotomy with or without femoral shortening and/or pelvic osteotomies (Salter and Pemberton) are most frequently used [1, 5, 21–23]. However complications like avascular necrosis and joint stiffness continue to be too high [8, 9, 22, 23]. In our case, we think that the lack of femoral shortening had led to joint stiffness and in this manner particularly contributed to the femoral neck fracture complication. Aydin et al. reported the results of Pemberton osteotomy in toddlers and preschool children and concluded that the avascular necrosis rate is parallel with the age and severity of dislocation [24]. Some authors reported that the additional femoral shortening decreases the complications of open reduction like joint stiffness and avascular necrosis [25, 26]. Shih et al. reported limited complications in 86 neglected DDH cases treated with one-stage correction by open reduction and Pemberton osteotomy with optional femoral shortening [22]. Zhao et al. compared simple Pemberton osteotomy and combined it with proximal femoral derotation-varization osteotomy for developmental dysplasia of the hip in groups aged 4–9 years and concluded that avascular necrosis and joint stiffness are less common in the combination group [27]. It is generally known that older children and the high dislocations are most likely to benefit from femoral shortening. However there is not a certain indication related with age and the degree of dislocation. Different reports are available in the literature. Good results are reported in patients older than 5 years of age, whereas Galpin et al. reported femoral shortening in all children older than 2 years [4, 28]. Wenger et al. have even advocated femoral shortening in certain children younger than 2 years [26]. Sankar et al. reported 72 DDH cases which were treated surgically, and femoral shortening osteotomy was done independent of patient age and radiographic displacement, but only if it was necessary to ease femoral head reduction. They concluded that patients older than 36 months and patients with superior displacement of the proximal femur more than 30% of pelvic width were more likely to require a femoral shortening osteotomy [29]. In our case the reduction of femoral head was not difficult, but the manual ballottement was unsatisfactory. We hesitated about femoral shortening but eventually decided not to do it. The moderate joint stiffness and femoral neck fracture during passive motion brought us to think that 2-3 mm manual ballottement after reduction may be beneficial for preventing joint stiffness. Further case series studies are needed to prove it.

In conclusion older children with neglected DDH are more likely to have joint stiffness after open reduction. If there is even a little doubt about joint stiffness during operation, one should not refrain from femoral shortening. Also passive motion under general anesthesia should be applied very carefully with fluoroscopic control.
